# Diagnostic performance of cardiovascular magnetic resonance native T1 and T2 mapping in pediatric patients with acute myocarditis

**DOI:** 10.1186/s12968-019-0550-7

**Published:** 2019-07-15

**Authors:** Matthew D. Cornicelli, Cynthia K. Rigsby, Karen Rychlik, Elfriede Pahl, Joshua D. Robinson

**Affiliations:** 1 Division of Pediatric Cardiology, Ann & Robert H Lurie Children’s Hospital of Chicago Northwestern University, 737 N. Michigan Avenue, Suite 1600 225 E Chicago Avenue, Box 21, Chicago, IL 60611 USA; 2 Department of Medical Imaging, Ann & Robert Lurie Children’s Hospital of Chicago, Chicago, IL USA; 3 Department of Pediatrics, Northwestern University, Chicago, USA; 4 Department of Radiology, Feinberg School of Medicine, Northwestern University, Chicago, USA; 5 Statistics Core, Ann and Robert H. Lurie Children’s Hospital of Chicago, Stanley Manne Children’s Research Institute, Chicago, IL USA

**Keywords:** Cardiovascular magnetic resonance, T1 mapping, T2 mapping, Extracellular volume, Myocarditis, Pediatrics

## Abstract

**Background:**

Multiple studies in adult patients suggest that tissue mapping performed by cardiovascular magnetic resonance (CMR) has excellent diagnostic accuracy in acute myocarditis, however, these techniques have not been studied in depth in children.

**Methods:**

CMR data on 23 consecutive pediatric patients from 2014 to 2017 with a clinical diagnosis of acute myocarditis were retrospectively analyzed and compared to 39 healthy controls. The CMR protocol included native T1, T2, and extracellular volume fraction (ECV) in addition to standard Lake Louise Criteria (LLC) parameters on a 1.5 T scanner.

**Results:**

Mean global values for novel mapping parameters were significantly elevated in patients with clinically suspected acute myocarditis compared to controls, with native T1 1098 ± 77 vs 990 ± 34 ms, T2 52.8 ± 4.6 ms vs 46.7 ± 2.6 ms, and ECV 29.8 ± 5.1% vs 23.3 ± 2.6% (all *p*-values < 0.001). Ideal cutoff values were generated using corresponding ROC curves and were for global T1 1015 ms (AUC 0.936, sensitivity 91%, specificity 86%), for global T2 48.5 ms (AUC 0.908, sensitivity 91%, specificity 74%); and for ECV 25.9% (AUC 0.918, sensitivity 86%, specificity 89%). While the diagnostic yield of the LLC was 57% (13/23) in our patient cohort, 70% (7/10) of patients missed by the LLC demonstrated abnormalities across all three global mapping parameters (native T1, T2, and ECV) and another 20% (2/10) of patients demonstrated at least one abnormal mapping value.

**Conclusions:**

Similar to findings in adults, pediatric patients with acute myocarditis demonstrate abnormal CMR tissue mapping values compared to controls. Furthermore, we found CMR parametric mapping techniques measurably increased CMR diagnostic yield when compared with conventional LLC alone, providing additional sensitivity and specificity compared to historical references. Routine integration of these techniques into imaging protocols may aid diagnosis in children.

## Background

Acute myocarditis is an inflammatory disease of the myocardium with variable presentations and clinical outcomes ranging from complete recovery to chronic heart failure, cardiac transplantation or death. Diagnosis is challenging as endomyocardial biopsy is invasive and prone to sampling error [1] and comprehensive and specific serologic testing remains elusive [2]. In the absence of a gold standard diagnostic test, cardiovascular magnetic resonance (CMR) has emerged as an important diagnostic tool [1, 3]. The Lake Louise Criteria (LLC) are the conventional guidelines used to help establish the diagnosis of myocarditis by CMR and focus on fulfilling 2 out of 3 positive criteria for evidence of hyperemia, edema, and myocardial necrosis/fibrosis [3]. Multiple studies, however, have shown that the diagnostic accuracy of the LLC varies and can be as low as 36%, motivating the desire for a more accurate and sensitive diagnostic tool [4–6].

More recently, adult studies suggest that newer CMR techniques specifically assessing the characteristics of the myocardium, including native T1 mapping, T2 mapping, and calculation of extracellular volume (ECV), are more sensitive for diagnosing myocarditis and may offer additional prognostic value [4, 5, 7–9]. As opposed to the LLC, where the myocardial signal intensity is generally assessed in a qualitative or semi-quantitative manner that is subject to viewer interpretation, mapping techniques fully quantify the myocardial signal across all parameters allowing for unbiased and reproducible assessments of the myocardium that can detect both global and regional/segmental disease patterns [9–12]. Adult studies demonstrate there are cases where CMR mapping is superior to LLC in diagnosing myocarditis [8, 11, 13, 14]. A recent systematic review and meta-analysis suggests mapping techniques provide high diagnostic accuracy and have the potential to replace classic elements of the LLC [15]. As such, recent CMR guidelines recommend the use of routine mapping in cases of suspected myocarditis [16].

CMR mapping techniques have not been systematically studied in children with myocarditis. One limiting factor may be the significant practice variations that exist across academic pediatric institutions, many of whom do not perform mapping in routine practice, though its utility has become more apparent in the past few years [17]. Further, the additional technical challenges of performing parametric mapping in children, such as heart rate variability and motion artifact, in addition to differences in clinical presentations, etiologies, and outcomes make it important to establish normative mapping values in pediatrics as these data may differ when compared with adults. Therefore, our study aimed to: 1. Compare differences in mapping values between pediatric patients with a clinical diagnosis of myocarditis versus healthy controls 2. Determine if mapping data can be used to successfully diagnose children with global and/or regional presentations of myocarditis and 3. Assess the diagnostic performance of mapping in conjunction with the conventional LLC in pediatric patients.

## Methods

### Patients

After obtaining approval by the Institutional Review Board at Ann & Robert H. Lurie Children’s Hospital of Chicago, a retrospective chart review was performed. Data from patients <21 years with a diagnosis of acute myocarditis and a clinical CMR study with native T1 and T2 performed from 2014 to 2017 were included. In the absence of a gold standard diagnostic test and similar to both a multi-center retrospective pediatric study [17] and a comprehensive adult meta-analysis [15], clinical diagnosis of acute myocarditis was used for study inclusion. Clinical diagnosis was determined by experienced clinicians and based on a presentation that included acute chest pain, viral respiratory symptoms, elevated troponin, recent infectious symptoms, and/or depressed systolic function on echocardiogram. All patients underwent CMR within 4 weeks of diagnosis of myocarditis.

CMR exams that included myocardial mapping data from age matched healthy pediatric subjects served as the control group. Typical control subjects were healthy patients who underwent CMR to assess for a coronary anomaly (*n* = 9) or cardiomyopathy/arrhythmogenic right ventricular cardiomyopathy (*n* = 30). All controls had normal cardiac structure and normal biventricular size and global systolic function determined by echocardiogram and CMR, no evidence of late gadolinium enhancement (LGE) determined by CMR, and no underlying systemic illnesses known to alter mapping values. Patients with a previous diagnosis of myocarditis, chronic cardiac disease, congenital heart disease, or an obvious alternative diagnosis (e.g. myocardial infarction) were excluded.

### CMR imaging protocol

All CMR studies were performed on a 1.5 T CMR scanner (Siemens Healthineers, Erlangen Germany). The CMR protocol was adapted from the previously published myocarditis consensus paper [3]. The protocol consisted of balanced steady state free precession (bSSFP) cine images in ventricular short- and long-axis views to assess for cardiac size and systolic function. Volumetric and functional data analysis was performed on dedicated software (Q Mass, Medis Medical Imaging Systems, Leiden, The Netherlands). T2-weighted (T2W) short-tau triple inversion-recovery (STIR) short axis to assess for edema, T1-weighted (T1W) turbo-spin-echo long axis imaging sequences pre and post contrast to assess for early gadolinium enhancement (EGE), and LGE were performed. Using the established LLC, myocardial edema was defined as a signal intensity ratio of myocardium relative to skeletal muscle ≥2.0 on T2W imaging [3]. We performed early gadolinium enhancement (EGE) imaging and LGE imaging 3 and 10–15 min, respectively. After the administration of contrast, 0.2 mmol/kg gadopentetate dimeglumine (Magnevist, Bayer HealthCare, Whippany, New Jersey, USA) or 0.15 mmol/kg of gadobutrol (Gadavist, Bayer HealthCare). EGE was defined as a signal intensity ratio of myocardium relative to skeletal muscle ≥4.0 on post-contrast images or an absolute increase in myocardial enhancement of ≥45% [3]. The presence of LGE was confirmed by at least two experienced observers at the time of the original study interpretation. General anesthesia was utilized as clinically necessary per our clinical protocol.

### CMR mapping

Myocardial native T1 maps were obtained using a free-breathing motion-corrected, electrocardiogram (ECG)-triggered, modified Look-Locker inversion recovery (MOLLI) sequence with images acquired at end-diastole before and 12 min after contrast injection in the short axis plane at the base, mid cavity, and apical LV myocardium, similar to previous protocols [18]. While acquisition times varied depending on the patient’s heart rate, 11 s was typically needed for each breath hold. Sequence parameters were optimized by adjusting the number of recovery heartbeats based on the patient’s heart rate to allow for adequate T1 recovery between inversion pulses (Table 1). In order to compensate for loss of spatial resolution due to cardiac motion at high heart rates, we optimized spatial resolution by reducing in-plane pixel size. Detailed T1 MOLLI parameters are provided in Table 1. T1 values were obtained using post-processing software (Medis Medical Imaging). Endocardial and epicardial contours were traced on each pre and post contrast image to exclude blood, epicardial fat, or artifact. The myocardium was divided into 16 segments as standardized by the American Heart Association (AHA) [19]. T1 values pre- and post-contrast for each myocardial segment were averaged to give a mean global T1 value. ECV was calculated using the following equation: ECV = λ × (1 − hematocrit), where the partition coefficient λ = ΔR1 (myocardium)/ΔR1 (blood) and ΔR1 is the change in signal intensity between pre- and post-contrast images [20].

**Table 1:** Typical Cardiovascular magnetic resonance (CMR) parameters for a) parametric mapping and b) T1 mapping number of recovery heartbeats

a)		
	T1 Mapping	T2 Mapping
Voxel Size	0.7 × 0.7 × 8.0 mm	1.9 × 1.9 × 8.0 mm
Temporal Resolution	311.25 ms	193.27 ms
Echo Time	1.03 ms	1.06 ms
Field of View Phase	85.2%	80%
Phase Resolution	196 × 256	116 × 192
Distance factor between slices	20%	20%
b)		
Heart Rate (bpm)	Number of Recovery Heart Beats Between Inversion Pulses for T1 Mapping	
60–70	5	
71–80	6	
81–90	7	
91–100	8	
101–110	9	
111–125	10	
125–140	11	

T2 mapping was performed using a dark blood turbo spin echo sequenced with a T2 preparation pulse and a bSSFP readout in the short-axis plane at the base, mid-chamber, and apex at end-diastole during free breathing with motion correction [21]. Detailed T2 imaging parameters are provided in Table 1. T2 mapping echo times were 0 ms, 24 ms, and 55 ms and did not vary by heart rate. Images were motion corrected. T2 maps were acquired prior to contrast administration. T2 values were obtained using post-processing software (Medis Medical Systems) and endocardial and epicardial contours were manually traced on each image to exclude blood, epicardial fat, or artifact. T2 values for each myocardial segment were recorded and each value was averaged to provide a mean global T2 value. Maximum segment T2 values were also recorded, similar to the methodology from adult studies [11, 22].

### Statistical analysis

All descriptive patient values are reported as percent distributions and continuous variables as mean +/− standard deviation or median values with interquartile ranges. Two sample independent t-tests were used to compare normally distributed data and Wilcoxon rank sum tests used for non-normal data. Receiver operating curves (ROC) were generated for T1, T2, and ECV and the Youden index was used to determine ideal upper limit cut-off values for distinguishing patients with myocarditis from healthy patients, with their own respective sensitivity and specificity. The predicted probabilities of logistic regression were used to create ROC curves of the combined mapping parameters. A *p*-value < 0.05 was considered statistically significant. Data were analyzed in SAS 9.4 (SAS Institute Inc., Cary, North Carolina, USA)***.***

## Results

### Patient demographics

A total of 23 patients with a clinical diagnosis of myocarditis and 39 healthy controls met the inclusion criteria. The median age at the time of CMR for the myocarditis patients was 15.2 years (range, 0.6–18.8) and for the controls was 16.3 years (range, 10.5–18.3, *p* = 0.054). CMR was performed on average 4 days (range, 1–26) after admission and all CMR studies were performed within 11 days of diagnosis aside from one (26 days). Patient demographic and clinical data are summarized in Table 2. On presentation, patients demonstrated arrhythmias in 17% of cases, required inotropes 26% of the time, and all but one patient (96%) had an elevated troponin. Of note, there was no difference in left ventricular ejection fraction by CMR between patients with myocarditis (54.5% ± 7.3) and controls (57.0% ± 4.8, *p*-value 0.17).

**Table 2:** Patients Demographics of controls and myocarditis patients

	Healthy control (*n* = 39)	Acute myocarditis (*n* = 23)	*p*-value
Median age at CMR (years)	15.1 (IQR 11.3–17.2)	16.3 (IQR 14.7–17.7)	0.054
Gender (% male)	69%	61%	0.50
ICU Admission	–	13/23 (56.5%)	–
Required Inotropes	–	6/23 (26.1%)	–
Treated with IVIG	–	12/23 (52.2%)	–
Presence of Arrhythmias	–	4/23 (17.4%)	–
Elevated Troponin	–	22/23 (96%)	–
Time from diagnosis to CMR (days)	–	4.5 (range, 1–26)	–
Required Anesthesia for CMR	4/39 (10.3%)	6/23 (26.1%)	< 0.001
Left Ventricular Ejection Fraction (%)	57.0 ± 4.8	54.5 ± 7.3	0.17
Indexed LVEDV (ml/m^2^)	92 ± 16	92 ± 23	0.89
Edema Present (T2W)	–	13/23 (57%)	–
Hyperemia Present (T1W)	–	3/23 (13%)	–
LGE Present	–	20/23 (86%)	–
Met LLC criteria	–	13/23 (57%)	–

Mean values listed unless otherwise specified. CMR: Cardiovascular Magnetic Resonance, IQR: Interquartile range, ICU: Intensive Care Unit, IVIG: Intravenous Immunoglublin,, LVEDV: Left ventricular end-diastolic volume, LGE: late gadolinium enhancement, LLC: Lake Louise Criteria

### Global and regional mapping values

**Fig. 1:**
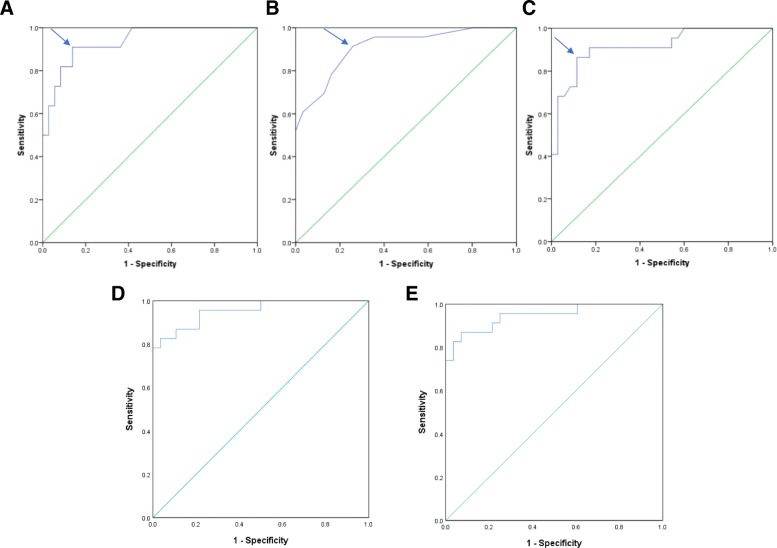
ROC Curves for CMR Mapping Parameters to Identify Patients with Acute Myocarditis. Receiver-operating curves (ROC) illustrate the performance of (**a**) global native T1, (**b**) global T2, and (**c**) extracelluar volume fraction (ECV). Ideal cut-off values for each mapping parameter were generated from each respective curve, (**d**) global native T1 mapping and global T2 mapping, and (**e**) global T1 mapping and maximum segmental T2 mapping (maxT2)

Mean global values for all mapping parameters were significantly elevated in patients with clinically suspected acute myocarditis compared to controls, with native T1 1098 ± 77 vs 990 ± 34 ms, T2 52.8 ± 4.6 ms vs 46.7 ± 2.6 ms, and ECV 29.8 ± 5.1% vs 23.3 ± 2.6% (all *p*-values < 0.001; Table 3). For each parameter, we established ideal cut off values from corresponding ROC curves to distinguish patients with myocarditis from controls, and to assess sensitivity and specificity. For global native T1 the ideal cutoff value was 1015 ms (AUC 0.936, sensitivity 91%, specificity 86%), for global T2 48.5 ms (AUC 0.908, sensitivity 91%, specificity 74%); and for ECV 25.9% (AUC 0.918, sensitivity 86%, specificity 89%) (Table 4, Fig. 1). Regional T2 mapping values were also significantly higher for patients with myocarditis as compared to controls. The maximum segmental T2 value for myocarditis patients was 61.2 ± 7.0 vs 52.4 ± 4.2 ms for controls (*p*-value < 0.0001). Finally, we created ROC curves using a combination of mapping parameters. Combining the global native T1 and global T2 parameters yielded an AUC 0.953 (sensitivity 83%, specificity 96%) while combining the global native T1 and maximum segmental T2 (MaxT2) parameters yielded an AUC of 0.947 (sensitivity 87%, specificity 93%) (Table 4, Fig. 1).

**Table 3:** CMR results comparing patients with acute myocarditis to controls

	Control (*n* = 39)	Acute myocarditis (*n* = 23)	*p*-value
Global native T1 (ms)	989.6 ± 34.3	1097.9 ± 77.0	<0.001
Global T2 (ms)	46.7 ± 2.6	52.8 ± 4.6	<0.001
ECV (%)	23.3 ± 2.4	29.8 ± 5.1	<0.001
Segmental maximum T2 (ms)	52.4 ± 4.2	61.2 ± 7.0	<0.001
Post-Contrast Global T1M (ms)	494.3 ± 53.4	475.6 ± 80.9	0.507

Mean values listed. ECV: extracellular volume

**Table 4:** Area Under the Curve (AUC) and Ideal Cut-off Values for the Three Major Mapping Values

	AUC	*p*-value	Ideal Upper Limit Cutoff Values	Sensitivity	Specificity
Global native T1	0.936	<0.0001	1015.5 ms	91%	86%
Global T2	0.908	<0.0001	48.5 ms	91%	74%
ECV	0.936	<0.0001	25.9%	86%	89%
Global native T1 + Global T2	0.953	<0.001	–	83%	96%
Global native T1 + MaxT2	0.947	<0.001	–	87%	93%

ECV: extracellular volume, MaxT2: Segmental maximum T2

### Combining LLC and mapping parameters

**Fig. 2:**
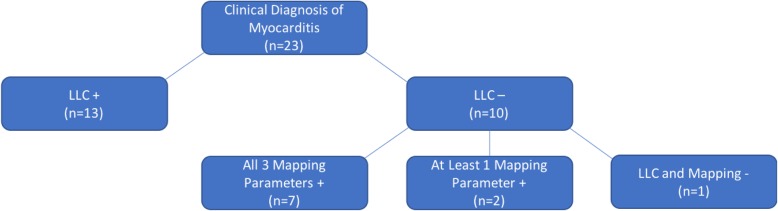
Cardiovascular Magnetic Resonance (CMR) Flowchart of Patients with a Clinical Diagnosis of Myocarditis. Applying generated cut-off values of each of the parametric mapping values (global native T1, global T2, and ECV) identified 90% of patients missed by the traditional Lake Louise Criteria (LLC). Only a single patient did not demonstrate positive findings by the LLC or by any of the mapping parameters

**Fig. 3:**
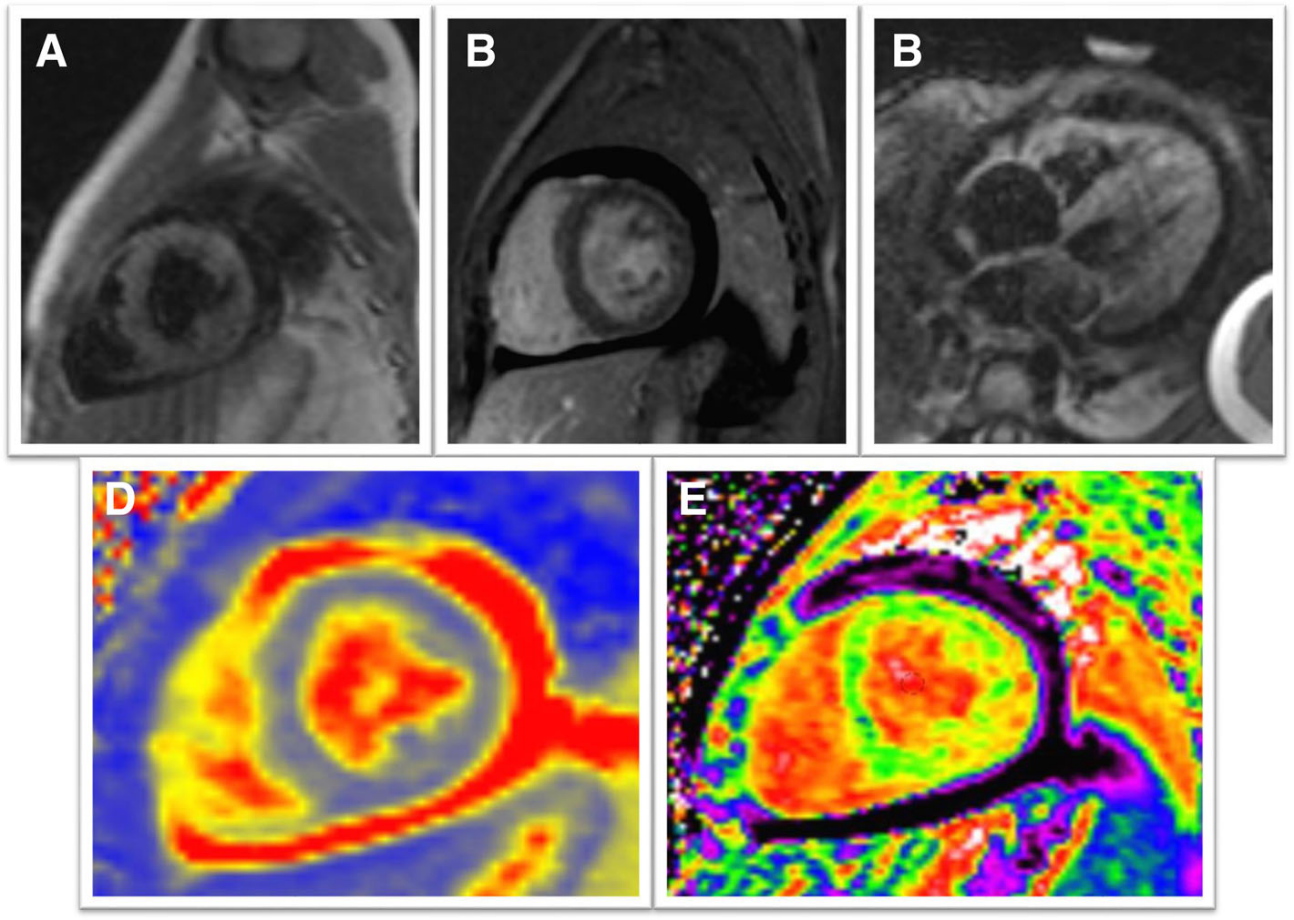
Selected cardiovascular magnetic resonance (CMR) images from a 1.5 year old girl with a clinical diagnosis of acute myocarditis. The top panel demonstrates normal selected images from the Lake Louise criteria (LLC), with: (**a**) no increased T2-weighted (T2W) signal intensity, (**b**) no evidence of late gadolinium enhancement (LGE), (**c**) no increased early gadolinium enhancement (EGE) and a pericardial effusion. The bottom panel demonstrates abnormal (**d**) T2M and (**e**) ECV maps in the mid-portion of the short-axis. The global native T1 was 1270 ms, the global T2 was 60 ms and ECV was 39.6%

We found only 57% (13/23) of patients with a clinical diagnosis of acute myocarditis met LLC. Broken down by each criterion, 86% had LGE, 57% had abnormal T2W imaging (edema), and 13% had abnormal EGE (hyperemia) (Table 2). Using the ROC curve generated cut-off values, ten patients with a clinical diagnosis were missed by the LLC. Of these ten, nine had at least one abnormal mapping value, seven demonstrated abnormal values across all three parameters, and two patients demonstrated abnormal maximum T2 values without any LGE (Fig. 2). Of particular interest, we identified two patients who demonstrated normal findings across all three of the LLC but who demonstrated abnormal mapping data across all three global mapping parameters. One of these patients exhibited markedly abnormal tissue mapping values, with global T1 1270 ms, global T2 60 ms, segmental max T2 of 66 ms and ECV 39.6%, and no perceptible LLC criteria (Fig. 3).

## Discussion

In this retrospective study we demonstrated that tissue mapping techniques can improve CMR diagnosis of acute myocarditis in pediatric patients. We established precise, reproducible normative mapping values in a pediatric population at our center, as evidenced by the low standard deviations seen in healthy children across all mapping techniques. Given the heterogeneous presentation of myocarditis, we examined both global and segmental mapping parameters and found significant differences in both for pediatric patients with myocarditis compared to controls. Finally, through an exploratory framework we generated AUC curves and ideal diagnostic cutoff values to demonstrate that mapping techniques in conjunction with the LLC may increase the sensitivity of CMR for the diagnosis of myocarditis in pediatric patients when compared with LLC alone. These findings are consistent with prior adult studies which were recently highlighted in a robust systematic review and meta-analysis [15].

Mean global native T1, T2, and ECV values were strong differentiators of pediatric patients with acute myocarditis compared to healthy controls, mirroring numerous studies published in the adult literature [4, 9, 12, 14, 22, 23]. While many of these studies examined patients that were either critically ill or had significant heart failure, we found important differences in global mapping values in a pediatric population that was generally less acutely ill—only a quarter of our patients required inotropic support and there was no significant difference in ejection fraction between controls and patients at the time of CMR. Though CMR referral practices may be different across adult and pediatric centers, a milder initial disease presentation appears more common in younger patients and similar to the findings of a previously published multi-center pediatric study [17]. Nonetheless, the strong performance of mapping techniques in our study, which included patients as young as 1.5 years old, may increase enthusiasm for obtaining CMR in younger, more critically ill patients.

Measuring segmental differences by T2 values can also identify adult patients with acute myocarditis by quantitatively assessing patchy and often subtle myocardial edema, which is thought to occur earlier than either changes in native T1 or LGE [11, 21, 22]. Our pediatric patients with myocarditis had significantly elevated maximum segmental T2 values when compared with controls. Moreover, two patients in our cohort demonstrated abnormal maximum segmental T2 values, yet did not have LGE on CMR, suggesting that T2 may help detect focal, and perhaps earlier disease patterns [23].

Perhaps most importantly, in addition to being sensitive across variable disease presentations, mapping techniques may address deficiencies in the conventional LLC observed in both pediatric and adult patients [4, 5, 7, 17, 24]. In our study only 57% of pediatric patients with a clinical diagnosis of myocarditis met the LLC. This limited sensitivity is consistent with previous studies, which reported the diagnostic accuracy of LLC at 36–83% in both pediatric and adult populations [3–6, 17, 25, 26]. These limitations have motivated intense inquiry into quantitative mapping techniques and adult studies have shown that such measures often outperform the LLC in diagnostic accuracy [5, 9, 11, 13, 15, 21, 22]. In this study of pediatric patients, our robust AUC, estimated sensitivities of 83–91% and specificities of 74–96% are similar to those published in the recent adult systematic meta-analysis [15]. Our findings showed that the presence of multiple abnormal mapping parameters further increased the AUC, suggesting improved specificity with good diagnostic yield. Moreover, we have highlighted multiple children who demonstrated abnormal mapping data and met none of the LLC criteria. Further multi-center study across pediatric centers will be necessary to establish the optimal combination of diagnostic mapping parameters for pediatric myocarditis.

Though the generalizability of our normative ranges for and diagnostic performance of these parametric mapping values is uncertain, this study adds to the growing evidence that parametric mapping should be incorporated into standard CMR protocol in cases of suspected myocarditis and is congruent with new recommendations for detecting myocarditis by CMR [27].

### Limitations

Our single center study using one CMR scanner is limited by a small sample size. The use of clinical criteria for the diagnosis is not an ideal substitute for a gold standard test but is a limitation of other similar diagnostic cohort and case control studies of myocarditis. Our study demonstrated overall lower CMR and global mapping values for healthy controls than previously published adult studies [8, 9, 15, 18, 21]. Some of this may due to the technical differences of obtaining CMR in children, who have more heart rate variability and often require anesthesia. Additionally, while consensus guidelines note these differences are expected, variable sequence parameters, post-processing techniques and vendor software, and possibly naturally occurring lower mapping values in children compared to adults all limit the generalizability of our data to other centers [5, 9, 10, 16].

## Conclusions

Similar to findings in adults, pediatric patients with acute myocarditis demonstrate abnormal CMR tissue mapping values compared to controls, s. Furthermore, mapping techniques measurably increased CMR diagnostic yield when compared with conventional LLC alone, providing additional sensitivity and specificity compared to historical references. Routine integration of these techniques into imaging protocols may aid diagnosis in children.

## Data Availability

The datasets used and/or analyzed during the current study are available from the corresponding author on reasonable request.

## Abbreviations

bSSFP: balanced steady state free precession
CMR: Cardiovascular magnetic resonance
ECV: Extracellular volume
EGE: Early gadolinium enhancement
LGE: Late gadolinium enhancement
LLC: Lake Louise criteria
MaxT2: Segmental maximum T2
MOLLI: (Modified Look-Locker Inversion Recovery)
ROC: receiver operator curve
STIR: short tau triple inversion recovery
T1W: T1-weighted
T2W: T2-weighted
